# Editorial: Bioremediation of Chemical Pesticides Polluted Soil

**DOI:** 10.3389/fmicb.2021.682343

**Published:** 2021-05-21

**Authors:** Xing Huang, Guining Lu

**Affiliations:** ^1^College of Life Sciences, Nanjing Agricultural University, Nanjing, China; ^2^School of Environment and Energy, South China University of Technology, Guangzhou, China

**Keywords:** bioremediation, chemical pesticides, microbial degradation, polluted soil, contamination

Since the discovery of certain synthetic organic pesticides in the 1940s, such as dichloro-diphenyl-trichloroethane (DDT) and hexachlorocyclohexane (BHC), many highly efficient chemical pesticides have been largely produced and are widely used to control field weeds, plant pathogens, and agricultural pests (Sharma et al., 2020). Pesticides play a vital role in the protection of crop yields, but their excessive and persistent use has resulted in severe environmental pollution and human health risks. Additionally, the residues of some herbicides could also cause severe phytotoxicity to rotation-sensitive crops (Huang et al., 2017). Therefore, the elimination of residual pesticides from polluted soils has attracted exclusive attention among researchers and the public. Based on numerous advantages, such as low cost, high removal efficiency, and environmental friendliness, microbial degradation has become a major metabolic pathway of chemical pesticides in the environment (Xu et al., 2019). Microorganisms capable of degrading various pesticides have been isolated and identified successfully from different environments (Arora, 2020; Morya et al., 2020). Bioremediation, the treatment that uses living organisms to transform hazardous substances into lesser or not-toxic compounds, is an effective way to clean soil polluted by chemical pesticides. In recent years, an increasing number of studies have gradually focused on the elucidation of the microbial mechanisms involved in the biodegradation and bioremediation of chemical pesticide contamination.

This Research Topic aims to provide a suitable occasion to publish updated research results regarding the bioremediation of chemical pesticide polluted soil by various functional microorganisms. This Research Topic consists of three research articles and four reviews.

Herbicide paraquat accumulation is emerging as a problem in an ever-growing portion of agricultural land. Inthama et al. isolated a paraquat-degrading bacterium, *Bacillus aryabhattai* MoB09, from paraquat-contaminated agricultural soil. This strain could not only remediate the paraquat residues in soil but also was responsible for promoting plant growth. In brief, strain MoB09, as a microorganism fertilizer or microbial agent, was expected to be applied for mitigating paraquat residue in an agricultural field. At the same time, Wu et al. isolated paraquat-transforming strain *Pseudomonas geniculata* PQ01 using an anaerobic enrichment procedure. Sucrose, glucose, pyruvate, formic acid, and acetic acid were shown to be favorable electron donors for the reduction of anthrahydroquinone-2,6-disulfonate (AQDS) reduction by PQ01, and the presence of sucrose significantly enhanced biotransformation. *P. geniculate* had much more potential applications in the field of environmental protection. This information might provide an in-depth understanding of the environmental functions of humic-reducing microorganisms involving inorganic pollutants.

Whittington et al. used a packed-bed microbial bioreactor to assess the role of the natural soil microbial communities during biodegradation of the triazolopyrimidine fungicide ametoctradin. The authors demonstrated the ability of a naturally sourced microbial consortium to degrade ametoctradin and generate metabolite profiles *in vitro*. The results of high-throughput sequencing showed that biodegradation of ametoctradin in both *ex vivo* soils and *in vitro* in the bioreactor correlated with an increase in the relative abundance of *Burkholderiales*, which is a genus of well-characterized microbial degraders of xenobiotic compounds.

Novel bioremediation methods are still waiting to be discovered. Jaiswal and Shukla provided an outlook of alternative strategies for bioremediation via synthetic biology. Mainly, this review presented how to utilize gene-editing tools, TALEN, CRISPR-Cas, and ZFNs, to obtain a host with target gene sequences that are responsible for the degradation of recalcitrant compounds. A genetic approach can increase the perspective of enzyme-based bioremediation. Notably, the ecological safety of bioremediation bacteria should be considered because it will be performed in an open environment rather than in a closed fermentation tank.

Zhang et al. summarized the latest reports regarding lindane biodegradation mechanisms and metabolic pathways by degrading microbes, as well as the microbial remediation technologies of lindane-contaminated soils, for instance, plant-microbe association and genetic engineering technologies. In addition, the migration and transformation of lindane in the soil were also analyzed. These novel understandings and bioremediation technologies aided the rapid removal of lindane from the soil and water system.

Long-term exposure to acephate and methamidophos in non-target organisms results in serious toxic impacts. Lin et al. reviewed the poisonous effects of acephate and methamidophos on aquatic and land animals. Their physicochemical degradation mechanisms and pathways were also explained. In this review, the mechanisms and degradation pathways of acephate and methamidophos were summarized to provide a new pathway for the study of acephate and methamidophos degradation.

Some neonicotinoid-degrading microorganisms, i.e., *Bacillus, Mycobacterium, Pseudoxanthomonas, Rhizobium*, and *Rhodococcus*, have been isolated and characterized. However, the microbial degradation mechanisms of neonicotinoids have rarely been reported. Pang et al. summarized the microbial degradation and biochemical mechanisms of several neonicotinoid insecticides. The potential of neonicotinoid-degrading microbes for the bioremediation of contaminated sites was also discussed. The detailed essential work still needs to be carried out before large-scale application of neonicotinoid-degrading microorganisms for the bioremediation of contaminated soil.

This Research Topic highlights that microorganisms play significant roles in the bioremediation of pesticide contaminated soil. Microbial consortia have a more dominant impact on the bioremediation of contaminated soils compared with a single strain. Plant-microbe associated bioremediation techniques are effective and cost-efficient methods of cleaning polluted sites, which is a promising method and could be used widely to significantly remove pesticides from the soil. Synthetic biology addresses the decontamination and remediation strategies for pesticides from the environment. Microbial synthetic biology remediation strategies not only increase the efficiency of microbial bioremediation processes for a particular contaminant but also provide the best methodologies for researchers. Given the health risks and the environmental impact of chemical pesticide polluted soils, microbial elimination-based bioremediation approaches, and associated technologies should be widely developed and applied in the future.

In summary, this Research Topic will expand our knowledge regarding the recent development in the field of bioremediation of chemical pesticides polluted soil. We sincerely hope more scientists will be inspired by our Research Topic to make contributions to tackle the global challenges caused by chemical pesticide pollution.

## Author Contributions

All authors contributed to the writing and approval of this editorial.

## Conflict of Interest

The authors declare that the research was conducted in the absence of any commercial or financial relationships that could be construed as a potential conflict of interest.
